# New Wenshen Shengjing Decoction Improves Early Embryonic Development by Maintaining Low Levels of H3K4me3 in Sperm

**DOI:** 10.1155/2022/9775473

**Published:** 2022-02-21

**Authors:** Wansheng Zhang, Lei Lu, Yanmei Sun, Zhu Dong, Xinyu Lei, Zhendong Peng, Baoyu Zhang, Shiwen Cao, Xuenan Wang, Xiaoyan Pan

**Affiliations:** ^1^Center for Reproductive Medicine, Jilin Medical University, Jilin 132013, China; ^2^Department of Urology Surgery, The Affiliated 465 Hospital of Jilin Medical University, Jilin 132013, China; ^3^Department of Neonatology, Jilin Central General Hospital, Jilin 132011, China; ^4^Reproductive Medicine Center of the Affiliated Hospital of Jining Medical College, Jining 272029, China

## Abstract

**Background:**

New Wenshen Shengjing Decoction (NWSSJD), a traditional Chinese compound medicine, has significant effect on spermatogenesis disorder and can significantly improve sperm quality. Many components in NWSSJD can induce epigenetic modifications of different types of cells. It is not yet known whether they can cause epigenetic modifications in sperm or early embryos.

**Objective:**

This study investigated the effect of NWSSJD on mouse early embryonic development and its regulation of H3K4me3 in mouse sperm and early embryos.

**Methods:**

Spermatogenesis disorder was induced in male mice with CPA (cyclophosphamide). NWSSJD was administrated for 30 days. Then, the male mice were mated with the female mice with superovulation, and the embryo degeneration rate of each stage was calculated. Immunofluorescence staining was used to detect the expression of H3K4me3 in sperm and embryos at various stages. Western blotting was performed to detect methyltransferase SETD1B expression. The expressions of development-related genes (*OCT-4*, *NANOG*, and *CDX2*) and apoptosis-related genes (*BCL-2* and *p53*) were measured with qRT-PCR.

**Results:**

Compared with the CPA group, NWSSJD significantly reduced the H3K4me3 level in sperms, significantly increased the number of normal early embryos (2-cell embryos, 3-4-cell embryos, 8-16-cell embryos, and blastocysts) per mouse, and reduced the degeneration rate of the embryos. The expression levels of H3K4me3 and methyltransferase SETD1B in early embryos were significantly elevated by NWSSJD. Additionally, NWSSJD significantly promoted *BCL-2* expression, while reducing *p53* expression, thus inhibiting embryonic cell apoptosis. Moreover, the expressions of development-related genes *OCT-4* and *CDX2* were significantly increased by NWSSJD, but *NANOG* expression had no significant difference.

**Conclusion:**

NWSSJD may promote early embryonic development possibly by maintaining low H3K4me3 levels in sperms and normal H3K4me3 modification in early embryos and by inhibiting embryonic cell apoptosis.

## 1. Introduction

The New Wenshen Shengjing Decoction (NWSSJD) is a traditional Chinese compound medicine and has been widely used to treat spermatogenesis disorders in males [1–5]. It is composed of 15 kinds of traditional Chinese herbs, mainly including ginseng, antler, Cynomorium, Astragalus, Epimedium, and Angelica [1]. Studies have found that NWSSJD can significantly increase the expressions of LHR (luteotropic hormone receptor) and P450scc (cholesterol side-chain cleavage enzyme) in the testis and promote the secretion of testosterone by the testicular tissue [1] and the development of seminiferous epithelia [4]. NWSSJD has a significant antioxidant effect [2], which can protect the testis and epididymis from oxidative damage by increasing the activities of GSH-Px (glutathione peroxidase) and CAT (catalase) in mature sperm and can significantly improve the activity of mitochondria in sperms [3]. It plays an important therapeutic role in oligoasthenospermia. A recent study has found that oligoasthenospermia is closely related to abnormal sperm epigenetic modification [6]. At present, most researches are focused on the effects of NWSSJD on spermatogenesis and sperm maturation. There is no report on the mechanism of NWSSJD on early embryonic development and epigenetic regulation.

Studies have found that the epigenetic modification of sperms can be passed to the early embryo and can regulate the development of the early embryos [7, 8]. Sperm chromatin is highly concentrated, containing 90%-99% protamine and 1%-10% histones. Among them, H3K4me3 (trimethylation of H3K4) is an important epigenetic modification, which plays an important regulatory role in the development of sperms and early embryos [9–12]. It is mainly distributed in the CpG-rich promoters [13] and participates in the regulation of gene transcription activation [14]. In histone demethylase KDM1A transgenic mice, H3K4me3 modification showed significant changes in sperms [10]. These changes were also observed in the paternal alleles of the preimplantation embryos, and there were nongenetic phenotypes in the offspring. Some environmental factors, such as the herbicide atrazine, can cause changes in sperm H3K4me3, which will cause the F1 and F3 generations of mice to have corresponding changes in H3K4me3 levels, and disrupt gene transcription level and spermatogenesis of offspring [15]. H3K4me3, as an important epigenetic modification of organism development, has gradually become an important target of drug therapy [16–19]. Our previous study found that NWSSJD could regulate the modification level of H3K9me2 in spermatocytes [5]. It is not yet known whether NWSSJD could affect the modification of H3K4me3 in sperms and early embryos.

Herein, a mouse model with spermatogenesis disorder was established by cyclophosphamide (CPA) induction. On the one hand, we studied the effect of NWSSJD on early embryo development. On the other hand, we explored the possible effect of NWSSJD on the epigenetic modification of H3K4me3 in sperm and early embryos. Our findings may shed light on the understanding of the treatment mechanism of NWSSJD on spermatogenesis disorder.

## 2. Materials and Methods

### 2.1. Animals

Kunming mice (10-week-old male mice, *n* = 225; 8-week-old female mice, *n* = 150) were from Yisi Laboratory Animal Technology Co., Ltd. (Changchun, China). The mice were kept in a temperature-controlled room (23 ± 2°C) with a light cycle of 12 h light (6:00-18:00)/12 h dark (18:00-6:00) and with free access to food and water. The animal experimental procedures were approved by the Ethics Committee of Jilin Medical University.

### 2.2. Preparation of NWSSJD

The components of NWSSJD included 6 g of ginseng, 9 g of Cynomorium, 12 g of Astragalus, 6 g of Epimedium, 1 g of velvet antler, 9 g of Cistanche, 6 g of Angelica, 9 g of Astragalus complanatus, 15 g of Chinese yam, 6 g of Atractylodes macrocephala, 3 g of Ligusticum wallichii, 6 g of Paeonia lactiflora, 1 g of Cinnamon, 1.5 g of Radix Aucklandiae, and 3 g of Fructus Foeniculi [1]. All the medicinal herbs were purchased from Tong Ren Tang, Beijing, China. According to the formula, each Chinese medicinal herb was weighed and prepared following the traditional Chinese medicinal decoction method [20]. Finally, the decoction with the concentration of 2 g crude medicinal herbs per mL was prepared and kept at 4°C for later use.

### 2.3. Toxicity Test of NWSSJD

According to the conversion of body surface area, the dose of NWSSJD for an adult mouse was calculated as 12 g·kg^−1^·d^−1^, which is 10 times that for human adults [21]. To test the toxicity of NWSSJD, male mice were divided into a control group (normal saline), clomiphene group (21.6 *μ*g·g^−1^·d^−1^) [22], low-dose NWSSJD group (6 g·kg^−1^·d^−1^), and high-dose NWSSJD group (12 g·kg^−1^·d^−1^), with 15 mice in each group. The treatment was administered by gavage once a day for 1 month. The food intake, water intake, behavior, and activity of mice were observed [23]. The weight gain and organ indexes, including the testis index, accessory gonad index, kidney index, spleen index and thymus index, were measured. The organ index was calculated as the ratio of organ weight (mg) to body weight (g) [23].

### 2.4. Spermatogenesis Disorder Model Establishment and Animal Treatment

Male mice were divided into the control group, NWSSJD group, NWSSJD+CPA group, and CPA group, with 55 mice in each group. Mice in the NWSSJD+CPA group and the CPA group were injected intraperitoneally with 80 mg/(kg·d) CPA (Tonghua Maoxiang Pharmaceutical Co., Ltd., China) for 5 consecutive days [24], and mice in the NWSSJD group and the control group were injected with an equal volume of normal saline. Then, the mice in the NWSSJD and NWSSJD+CPA groups were intragastrically administrated with NWSSJD at a dose of 12 g·kg^−1^·d^−1^ for 30 days. The control group and the CPA group were administrated with an equal volume of normal saline for 30 days. Five mice from each group were sacrificed to collect sperms. The remaining mice of each group mated with the superovulated female mice.

### 2.5. Sperm Collection

Male mice were sacrificed by cervical dislocation. The epididymides on both sides were quickly removed and placed in a petri dish containing 1 mL of 37°C normal saline. After ripping the epididymal tail with a 26-gauge needle, the epididymal tail was incubated at 37°C for 10 min to fully free the sperms from the epididymal tail.

### 2.6. Immunofluorescence Staining of Sperm

Sperm smear was prepared and then incubated with a sperm deagglutination solution (25 mM DTT, 0.2% Triton X-100, and 200 IU/mL heparin) at 37°C for 15 min. After that, the sperm smear was fixed in 3.7% paraformaldehyde for 20 min. Then, the sperm smear was washed with PBS and blocked with 5% BSA for 2 h at room temperature. After washing, the sperm smear was subjected to incubation with rabbit anti-H3K4me3 (A2357, ABclonal) for 2 h and then with goat antirabbit FITC-labeled secondary antibody (AS011, ABclonal) for 1 h. Hoechst 33342 (14533, Sigma) was used to stain the sperm nucleus. Five mice from each group were used for sperm smear, and 5 sperm smears were prepared from each mouse. The sperm smear was observed under the Olympus IX-53 (Olympus, Tokyo, Japan). Five high-power fields (×1000) were randomly selected. The microscopic image acquisition system (CellSens Dimension) was used to take photographs. ImageJ software (NIH, V1.8.0) was used to analyze the average fluorescence intensity of H3K4me3 in sperms.

### 2.7. Collection of Embryos

Female mice were subjected to superovulation. In detail, 10 IU pregnant horse serum gonadotropin was injected intraperitoneally, and 10 IU human chorionic gonadotropin (HCG) was injected 48 h later. Then, the female mice were caged with male mice at 1 : 1. The 2-cell embryos, 3-4-cell embryos, 8-16-cell embryos, and blastocysts were obtained from mouse fallopian tubes or the uterus at 42 h, 52 h, 70 h, and 94 h after HCG injection. They were observed under the Olympus IX-83 (Olympus, Tokyo, Japan). The embryo degeneration rate at each stage was calculated by the formula of (the number of degenerated embryos)/(the number of total collected embryos each time).

### 2.8. Immunofluorescence Staining of Embryos

The obtained embryos were fixed in 3.7% paraformaldehyde for 20 min and then blocked in 5% BSA for 1 h. After blocking, the embryos were incubated with rabbit anti-H3K4me3 (A2357, ABclonal) primary antibody for 2 h. After washing three times with PBS, goat antirabbit FITC-labeled secondary antibody (AS011, ABclonal) was added for incubation for 1 h. After staining the nucleus with Hoechst 33342 (14533, Sigma), the embryos were mounted and observed by a laser scanning confocal microscope (FV1000, Olympus, Tokyo, Japan). All pictures were taken under the same intensity of laser irradiation. The average fluorescence intensity of H3K4me3 of 20 embryos from three mice in each group was analyzed by ImageJ software (NIH, V1.8.0).

### 2.9. Western Blotting

Total proteins were extracted from the 2-cell embryos (*n* = 60 each time), 8-cell embryos (*n* = 20 each time), and blastocysts (*n* = 10 each time). After electrophoresis, proteins were transferred to the PVDF membrane. The membrane was blocked with 5% skimmed milk for 1 h and then incubated with rabbit anti-SETD1B (A20155, ABclonal) and anti-Lamin A/C (A0249, ABclonal) primary antibodies at 4°C overnight. After washing, the membrane was incubated in goat antirabbit HRP-labeled secondary antibody (AC028, ABclonal) for 2 h at room temperature. The color development was performed using an enhanced chemiluminescence reagent. The films were scanned using the ChemiDOC XRS+ imaging systems (Bio-Rad Laboratories, Hercules, CA, USA). ImageJ (NIH, V1.8.0) analyzed the relative expression level of SETD1B.

### 2.10. Quantitative Real-Time RT-PCR (qRT-PCR)

RNAs were extracted from blastocysts (*n* = 10 each time) using the RNeasy Micro Kit (QIAGEN, Hilden, Germany). The RNAs were reverse transcribed into cDNA in a 20 *μ*L reverse transcription system. The qRT-PCR was performed with the iQ5 Multicolor Real-Time PCR Detection System (Bio-Rad) and SYBR Premix Ex Taq (Takara Dalian, China). The primers used are shown in Table 1. The PCR reaction conditions were as follows: predenaturation at 95°C for 30 s, 40 cycles of denaturation at 95°C for 5 s, annealing at 60-62°C for 20 s, and extension at 72°C for 30 s. The housekeeping gene *β*-actin was used as an internal reference. The relative expression level of the target gene was calculated using the 2^-△△Ct^ method.

### 2.11. Statistical Analysis

The SPSS 17.0 software was used for statistical analysis. The data are expressed as mean ± standard deviation (SD). One-way ANOVA followed by the LSD post hoc test was used to compare differences among multiple groups. *P* < 0.05 was considered statistically significant.

## 3. Results

### 3.1. NWSSJD Has No Obvious Toxic and Side Effects on Mice

The toxicity of NWSSJD to mice was assessed. Compared with the control group, the food intake, water intake, behavior, and activity of mice in clomiphene group, low-dose NWSSJD group (6 g·kg^−1^·d^−1^), and high-dose NWSSJD group (12 g·kg^−1^·d^−1^) showed no obvious differences (Table 2). The weight gain and spleen index of mice in the clomiphene group were significantly lower than those in the control group (*P* < 0.05), but there was no significant difference in the testicular index, accessory gonad index, kidney index, and thymus index between the clomiphene group and the control group (*P* > 0.05). The weight gain, testicular index, accessory gonad index, kidney index, spleen index, and thymus index of the low-dose NWSSJD group and the high-dose NWSSJD group were not significantly different from those of the control group (*P* > 0.05). The results indicate that NWSSJD is basically nontoxic to mice.

### 3.2. NWSSJD Maintains the Low Expression Level of H3K4me3 in Sperm

**Figure 1:**
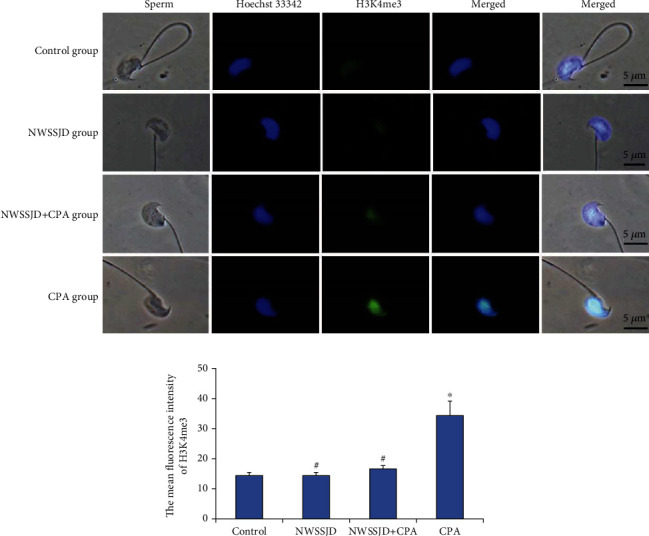
Expression of H3K4me3 in mouse sperm. (a) Immunofluorescence labeling of H3K4me3 in mouse sperm. H3K4me3 is marked in green, and the sperm nucleus is marked in blue. Scale bar: 5 *μ*m. (b) The average fluorescence intensity of H3K27me3 in sperm was measured with ImageJ software (*N* = 5). Compared with control, ^∗^*P* < 0.05. Compared with CPA, ^#^*P* < 0.05.

Mature sperms were obtained from the epididymal tail of mice in the control group, NWSSJD group, NWSSJD+CPA group, and CPA group, and the sperms were subjected to H3K4me3 immunofluorescence staining (Figure 1(a)). H3K4me3 was present in the nucleus of the sperm (Figure 1(a)). CPA induced significantly higher H3K4me3 levels than the control (Figure 1(b)). There was no significant difference in the average fluorescence intensity of sperm H3K4me3 among the NWSSJD group, the NWSSJD+CPA group, and the control group, while the average fluorescence intensity of sperm H3K4me3 in the NWSSJD group and the NWSSJD+CPA group was significantly lower than that in the CPA group (*P* < 0.05, Figure 1(b)). Thus, NWSSJD could maintain the low expression level of H3K4me3 in sperms.

### 3.3. NWSSJD Helps Maintain the Normal Development of Early Mouse Embryos

**Figure 2:**
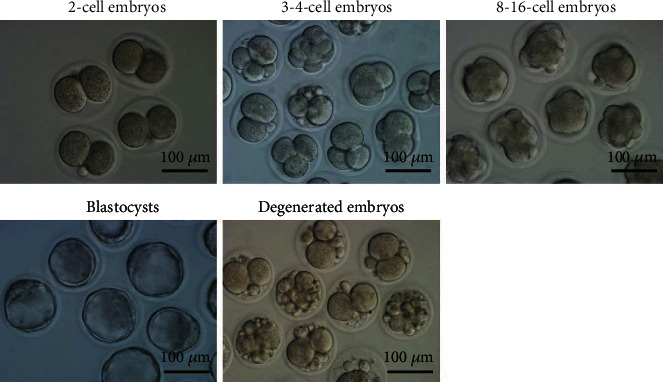
Morphology of early mouse embryos. The figure shows the representative morphology of 2-cell embryos, 3-4-cell embryos, 8-16-cell embryos, blastocysts, and degenerated embryos. Scale bar: 100 *μ*m.

At 42 h, 52 h, 70 h, and 94 h after HCG injection, 2-cell embryos, 3-4-cell embryos, 8-16-cell embryos, and blastocysts were obtained from mouse fallopian tubes or uterus, and the embryo degeneration rate of each stage was calculated. Embryos with blastomere fragmentation, vacuolization, and delayed development were considered to be degenerated embryos (Figure 2). Compared with the control group, CPA significantly reduced the number of 2-cell embryos, 3-4-cell embryos, 8-16-cell embryos, and blastocysts per mouse (*P* < 0.05) and increased the embryo degeneration rate of each stage (*P* < 0.05) (Table 3). The number of normal embryos obtained in each mouse and the embryo degeneration rate in each stage of the NWSSJD group and the NWSSJD+CPA group were not significantly different from those of the control group (*P* > 0.05) but were significantly higher than those of the CPA group (*P* < 0.05). This result indicates that the normal development of early mouse embryos could be maintained by NWSSJD.

### 3.4. NWSSJD Maintains the Normal Expression of H3K4me3 in Mouse Early Embryos

**Figure 3:**
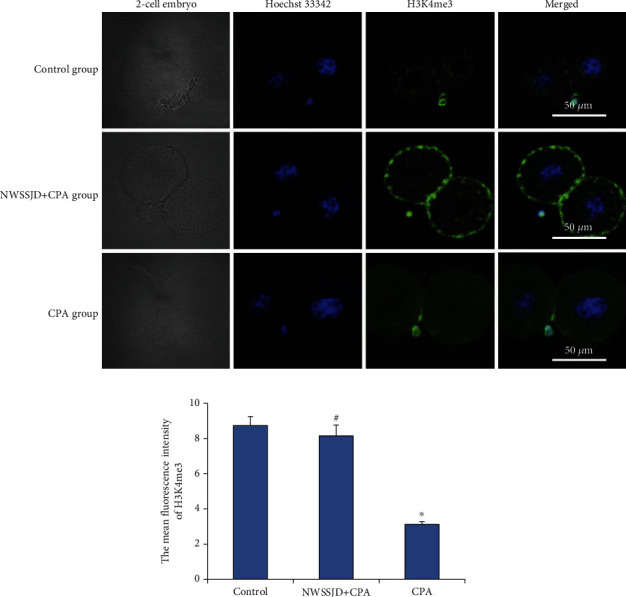
Expression of H3K4me3 in mouse 2-cell embryos. (a) Immunofluorescence labeling of H3K4me3 in mouse 2-cell embryos. H3K4me3 is marked in green, and the nucleus is marked in blue. Scale bar: 50 *μ*m. (b) The average fluorescence intensity of H3K27me3 in 2-cell embryos was measured with ImageJ software (*N* = 3). Compared with control, ^∗^*P* < 0.05. Compared with CPA, ^#^*P* < 0.05.

**Figure 4:**
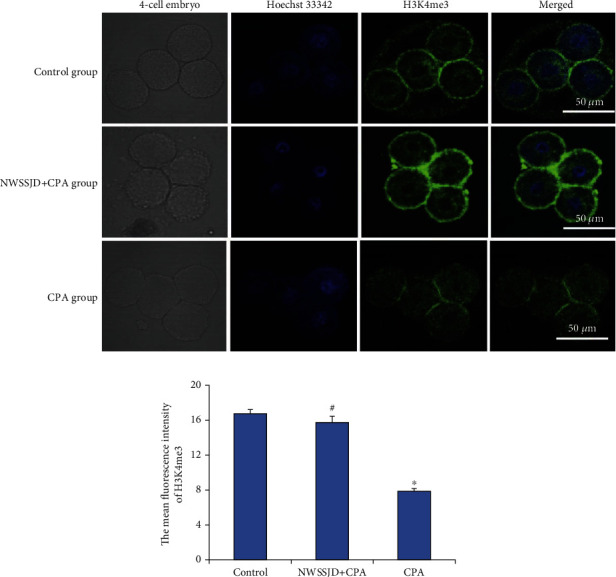
Expression of H3K4me3 in mouse 4-cell embryos. (a) Immunofluorescence labeling of H3K4me3 in mouse 4-cell embryos. H3K4me3 is marked in green, and the nucleus is marked in blue. Scale bar: 50 *μ*m. (b) The average fluorescence intensity of H3K27me3 in 4-cell embryos was measured with ImageJ software (*N* = 3). Compared with control, ^∗^*P* < 0.05. Compared with CPA, ^#^*P* < 0.05.

**Figure 5:**
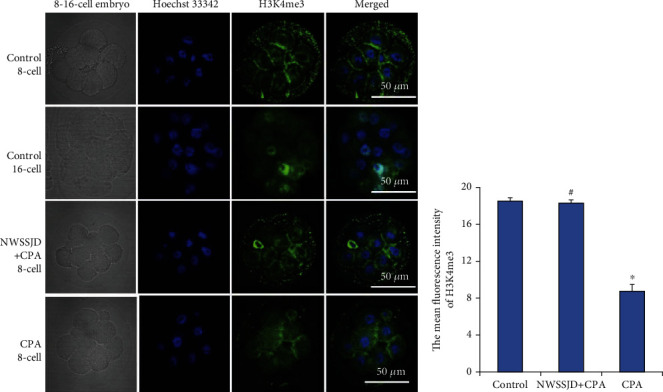
Expression of H3K4me3 in mouse 8-16-cell embryos. (a) Immunofluorescence labeling of H3K4me3 in mouse 8-16-cell embryos. H3K4me3 is marked in green, and the nucleus is marked in blue. Scale bar: 50 *μ*m. (b) The average fluorescence intensity of H3K27me3 in 8-16-cell embryos was measured with ImageJ software (*N* = 3). Compared with control, ^∗^*P* < 0.05. Compared with CPA, ^#^*P* < 0.05.

**Figure 6:**
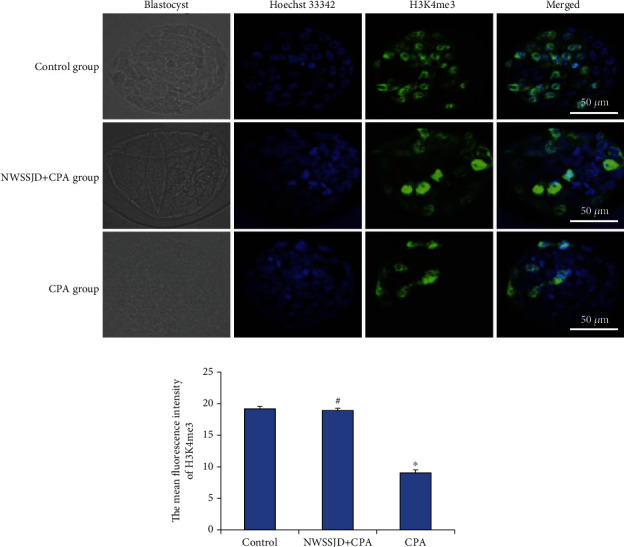
Expression of H3K4me3 in mouse blastocysts. (a) Immunofluorescence labeling of H3K4me3 in mouse blastocysts. H3K4me3 is marked in green, and the nucleus is marked in blue. Scale bar: 50 *μ*m. (b) The average fluorescence intensity of H3K27me3 in blastocysts was measured with ImageJ software (*N* = 3). Compared with control, ^∗^*P* < 0.05. Compared with CPA, ^#^*P* < 0.05.

H3K4me3 immunofluorescence staining was performed on the 2-cell embryos, 4-cell embryos, 8-16-cell embryos, and blastocysts collected in each group (Figures 3(a), 4(a), 5(a), and 6(a)), and the average fluorescence intensity of H3K4me3 in each stage of embryos was calculated (Figures 3(b), 4(b), 5(b), and 6(b)). It was found that the expression of H3K4me3 in 2-cell embryos, 4-cell embryos, and 8-cell embryos was mainly distributed in the perinuclear region. Starting from the 16-cell embryo stage, H3K4me3 distribution was transferred from the perinuclear region into the nucleus. In the blastocyst stage, the H3K4me3 expression in the nucleus was enhanced. Compared with the control group, CPA significantly reduced the expression of H3K4me3 in 2-cell embryos, 4-cell embryos, 8-16-cell embryos, and blastocysts (*P* < 0.05). The expression of H3K4me3 in embryos at each stage of the NWSSJD+CPA group was not significantly different from that of the control group, but it was significantly higher than that of the CPA group (*P* < 0.05). Therefore, NWSSJD could maintain the normal expression of H3K4me3 in early embryos.

### 3.5. NWSSJD Sustains the Normal Expression of Methyltransferase SETD1B in Mouse Early Embryos

**Figure 7:**
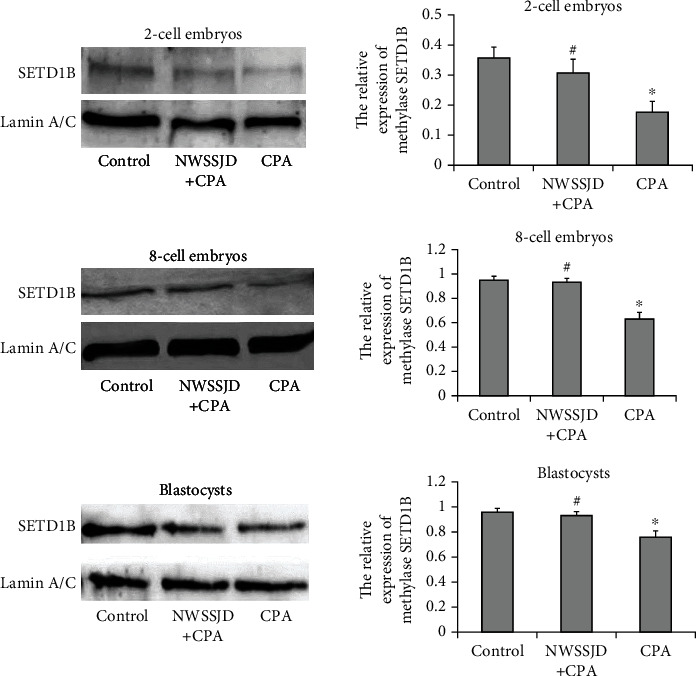
Expression of methyltransferase SETD1B in embryos. Western blotting detected the expression of methyltransferase SETD1B in 2-cell embryos, 8-cell embryos, and blastocysts. ImageJ software analyzed the relative expression level of SETD1B. Compared with control, ^∗^*P* < 0.05. Compared with CPA, ^#^*P* < 0.05.

The 2-cell embryos, 8-cell embryos, and blastocysts collected in each group were subjected to Western blotting analysis of methyltransferase SETD1B (Figure 7). Compared with the control group, CPA significantly reduced the expression of SETD1B in 2-cell embryos, 8-cell embryos, and blastocysts (*P* < 0.05). There was no significant difference in the expression of SETD1B in the embryos of each stage in the NWSSJD+CPA group compared with the control group. However, the SETD1B level in the embryos of each stage in the NWSSJD+CPA group was significantly higher than that in the CPA group (*P* < 0.05). This result indicates that NWSSJD could maintain the normal expression of the methyltransferase SETD1B in early embryos.

### 3.6. NWSSJD Increases the Expressions of Development-Related Genes and May Reduce the Embryonic Cell Apoptosis by Regulating the Apoptosis-Related Genes in Mouse Blastocysts

**Figure 8:**
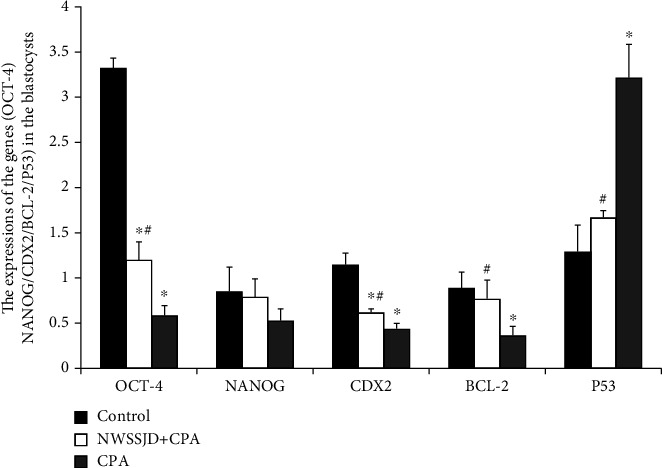
qRT-PCR analysis of the expression of development-related genes (*OCT-4*, *NANOG*, and *CDX2*) and apoptosis-related genes (*BCL-2* and *p53*) in blastocysts. Compared with control, ^∗^*P* < 0.05. Compared with CPA, ^#^*P* < 0.05.

The mouse blastocysts collected in each group were subjected to qRT-PCR analysis of genes related to embryonic development and differentiation (*OCT-4*, *NANOG*, and *CDX2*) and apoptosis-related genes (*BCL-2* and *p53*) (Figure 8). The results showed that, compared with the control group, CPA significantly reduced the expression of *OCT-4*, *CDX2*, and *BCL-2* and significantly increased the expression of *p53* (*P* < 0.05). Compared with the CPA group, NWSSJD significantly increased the expression of *BCL-2* and significantly reduced the expression of *p53* (*P* < 0.05). The expression of *BCL-2* and *p53* in the NWSSJD+CPA group was not significantly different from that in the control group. The expressions of *OCT-4* and *CDX2* in the NWSSJD+CPA group were significantly lower than those in the control group (*P* < 0.05). CPA and NWSSJD did not significantly affect the *NANOG* gene expression. This data suggests that NWSSJD could increase the expression of embryonic development-related genes and possibly reduce the embryonic cell apoptosis through regulating the expression of apoptosis-related genes.

## 4. Discussion

Traditional Chinese compound medicines contain a variety of Chinese herbal medicines with complex components, which may induce toxic effects [25]. Thus, the toxicity test of traditional Chinese compound medicines is very important [26]. In this study, we evaluated the toxicity of NWSSJD to mice. Our results showed that after NWSSJD administration, the daily activities of mice were normal compared with the control group, and there was no significant change compared with the control group in terms of weight gain and organ indexes, indicating that NWSSJD is basically nontoxic to mice.

NWSSJD can significantly promote the secretion of testosterone by testicular stromal cells, promote the development of testicular seminiferous epithelia, and inhibit the apoptosis of spermatogenic cells and sperms, which has a significant therapeutic effect on male oligoasthenospermia [1–5]. In this article, we demonstrated a new role of NWSSJD. Our results showed that NWSSJD could maintain the low modification level of H3K4me3 in sperms and the normal expressions of methyltransferase SETD1B and H3K4me3 in embryos and promote early embryo development and may inhibit embryonic cell apoptosis. This study provides a theoretical basis for further elucidating the treatment mechanism of NWSSJD.

Oligoasthenospermia is an important cause of male infertility. The etiology of oligoasthenospermia is complicated. For example, radiation exposure, chemotherapy drugs, or environmental endocrine disrupting agents can all cause oligoasthenospermia [8, 27]. Studies have found that these factors can not only cause oligoasthenospermia but also interfere with the epigenetic modification in sperms [28, 29]. These changed epigenetic modifications may even pass to offspring [30]. CPA is an immunosuppressant, which is widely used to treat a variety of autoimmune diseases and tumors [8]. Studies have found that CPA not only reduces the number of sperms in semen [31] but also damages the development of Leydig cells [32], disrupts the replacement of histones by protamine in sperm [33], and results in incomplete sperm chromatin structure and disordered epigenetic modification [30], which changed the expression pattern of acetylated H4K5 in 2-cell embryos [30]. H3K4me3 is an important modification of mature sperm histones, which can activate gene transcription [10]. The chromatin in the mature sperm is highly concentrated, and most genes are in a transcriptional repression state. Thus, the expression level of H3K4me3 is very low [10] in mature sperms. It is found that some drugs can alter the modification level of H3K4me3 in sperm, which may be closely related to male infertility [34]. Studies have found that epigenetic modifications of genetic materials from the paternal or maternal line will be reprogrammed after the formation of early embryos [35], and abnormal histone modifications from sperms or oocytes may cause abnormal histone modifications in early embryos [29]. In this study, we found that CPA caused abnormal H3K4me3 modification in sperms and early embryos, while NWSSJD maintained the normal H3K4me3 modification level in sperms and early embryos, which was not significantly different from the control group. Methyltransferase SETD1B can catalyze the trimethylation of H3K4 [36]. This study found that the expression level of SETD1B in the embryos of the CPA group was significantly reduced, while that in the NWSSJD+CPA group was not significantly different from that in the control group. This indicates that NWSSJD may maintain the normal H3K4me3 modification level through maintaining the normal expression level of SETD1B.

H3K4me3 regulates the expression of important genes during early embryonic development, such as *OCT-4*, *NANOG, SOX2*, and *CDX2* [11, 12, 37, 38], and the expression of apoptosis-related genes (*Bax*, *Bak*, and *Bcl*) [39]. Therefore, H3K4me3 exhibits different modification patterns in different stages of embryonic development [11, 12, 37, 38]. Before the activation of the zygotic genome, the H3K4me3 modification from the paternal chromosome will undergo extensive reprogramming. After the activation of the zygotic genome, the peak of H3K4me3 appears. Together with H3K27me3, H3K4me3 regulates the expression of genes related to embryonic development [40]. Wu et al. found that the expression level of H3K4me3 was lower in embryos before the 8-cell stage in cattle embryos and that from the 16-cell stage to the blastocyst stage, the expression level of H3K4me3 increased significantly [41]. In this study, we performed immunofluorescence staining of H3K4me3 on mouse embryos at various developmental stages and found a similar pattern. In mouse embryos at the 2-cell, 4-cell, and 8-cell stage, H3K4me3 was expressed in the perinuclear region of the blastomere, and the expression level was low. Starting from the 16-cell stage, H3K4me3 began to enter the nucleus, and the expression level was significantly increased. The main reason may be that with the improvement of the embryonic development stage, cell division and differentiation become more active, and more genes are needed to be activated. Therefore, the increase of the H3K4me3 level makes the chromosome in a more open state, which is beneficial to the expression of development-related genes (*OCT-4*, *NANOG*, and *CDX2*) and beneficial to early embryonic development. Zhang et al. used MM-102 to reduce the modification level of H3K4me3 in pig embryos with somatic cell nuclear transfer and found that the expression level of the apoptosis-related gene BCL-2 also decreased [42]. They suggest that H3K4me3 may be an important target molecule that regulates early development. Similarly, Wang et al. found that, by regulating the expression of miR-34c in donor cells, the expression levels of H3K4me3 in nuclear-transferred bovine 8-cell embryos were increased significantly, embryonic cell apoptosis was reduced, and the rate of cleavage and blastocyst development was increased [43]. Our study found that the NWSSJD+CPA group significantly increased the H3K4me3 modification level and the expression of *OCT-4*, *NANOG*, and *Bcl-2* genes in embryos than the CPA group. These results suggest that NWSSJD may increase the modification level of H3K4me3 in embryos, promote the expression of development-related genes, and regulate the expression of apoptosis-related genes, so as to reduce embryonic cell apoptosis and promote early mouse embryonic development.

Our lab has identified some of the active components in NWSSJD, including ginsenoside, icariin, limozine, velvet antler polypeptide, and other components (data not yet published). Studies have shown that ginsenoside Rg3 can regulate H3K14/K9 and H4 K12/K5/K16 acetylation modification in ovarian cancer cells [44], and limozine can cause changes in histone H3 and H4 acetylation and H3K27me3 modification in neural stem cells and cardiomyocytes [45, 46]. However, there is no report concerning the effect of NWSSJD on epigenetic modifications of sperms and early embryonic cells. For the first time, we found the regulation of NWSSJD on the levels of H3K4me3 in sperms and early embryos in male mice treated with CPA. The focus of our follow-up work is to determine which active components in NWSSJD regulate H3K4me3 in sperms or early embryos and other possible epigenetic modifications regulated by NWSSJD to further clarify the mechanism of NWSSJD.

Epigenetic modification is a controllable and reversible process. Several drugs targeting epigenetic regulation of diseases have been approved by USA FDA [47–49]. The protective effects of NWSSJD on male spermatogenesis disorders have been reported [1, 2, 4]. In our previous study, we found that the methylation modifications of sperm histones in mice with spermatogenesis disorders were abnormal, which were well improved after NWSSJD treatment [5, 50]. This suggests that NWSSJD may also be applied to patients with epigenetic modifications in sperms caused by environmental factors or chemical drugs, thus expanding the clinical application of NWSSJD. Additionally, the histone methylation modification of embryos returned to normal after NWSSJD treatment [50], which further confirms that NWSSJD may be a good targeted drug for sperm histone modification.

## 5. Conclusion

In summary, we found that NWSSJD can maintain the normal modification pattern of H3K4me3 in embryonic cells by maintaining a low level of H3K4me3 modification in sperms and improve the number of normal embryos per mouse. This study broadens our understanding of the treatment mechanism of NWSSJD and lays the foundation for promoting the clinical application of NWSSJD.

## Figures and Tables

**Table 1:** The sequence of primers used in qRT-PCR.

Gene	Primer sequences (5′ to 3′)		GenBank accession no.
	Forward	Reverse	
*OCT-4*	AGAGGATCACCTTGGGGTACA	CGAAGCGACAGATGGTGGTC	*NM_001252452*
*NANOG*	TCTTCCTGGTCCCCACAGTTT	GCAAGAATAGTTCTCGGGATGAA	*71950*
*CDX2*	CAAGGACGTGAGCATGTATCC	GTAACCACCGTAGTCCGGGTA	*12591*
*BCL-2*	GAGAGCGTCAACAGGGAGATG	CCAGCCTCCGTTATCCTGGA	*NM_1774102*
*p53*	ATGCCCATGCTACAGAGGAG	AGACTGGCCCTTCTTGGTCT	*NM_001127233*
*β-Actin*	TGTTACCAACTGGGACGACA	CTGGGTCA TCTTTTCACGGT	*BC138614.1*

**Table 2:** Effects of NWSSJD on body weight gain and organ index (*N* = 15).

Groups	Weight gain (mg/g)	Testicular index (mg/g)	Accessory gonad index (mg/g)	Kidney index (mg/g)	Spleen index (mg/g)	Thymus index (mg/g)
Control group	3.13 ± 0.18	5.81 ± 0.17	8.34 ± 0.27	12.57 ± 0.45	3.53 ± 0.60	1.57 ± 0.15
Clomiphene group	2.07 ± 0.23^∗^	5.48 ± 0.23	7.99 ± 0.54	12.40 ± 0.56	2.73 ± 0.31^∗^	1.33 ± 0.21
Low-dose NWSSJD group	3.11 ± 0.27	5.61 ± 0.18	8.18 ± 0.38	12.50 ± 0.56	2.87 ± 0.35	1.50 ± 0.20
High-dose NWSSJD group	3.11 ± 0.20	5.67 ± 0.14	8.37 ± 0.17	12.47 ± 0.67	3.37 ± 0.32	1.53 ± 0.12

Note: compared with control, ^∗^*P* < 0.05.

**Table 3:** The effect of NWSSJD on early embryonic development.

Groups	No. of 2-cell embryos per mouse	No. of degenerated 2-cell embryos per mouse	Rate of degenerated 2-cell embryos (%)	No. of 3-4-cell embryos per mouse	No. of degenerated 3-4-cell embryos per mouse	Rate of degenerated 3-4-cell embryos (%)	No. of 8-16-cell embryos per mouse	No. of degenerated 8-16-cell embryos per mouse	Rate of degenerated 8-16-cell embryos (%)	No. of blastocysts per mouse	No. of degenerated blastocysts per mouse	Rate of degenerated blastocysts (%)
Control group	19.4 ± 3.9	5.0 ± 1.6	22.2 ± 2.2	14.0 ± 2.7	1.8 ± 0.4	11.8 ± 4.0	12.4 ± 1.8	2.0 ± 1.6	12.5 ± 8.7	9.6 ± 1.8	0.8 ± 0.8	6.6 ± 6.6
NWSSJD group	17.2 ± 3.0^#^	4.6 ± 1.1^#^	20.9 ± 1.6^#^	14.8 ± 1.3^#^	1.8 ± 0.4^#^	10.9 ± 2.7^#^	12.8 ± 1.3^#^	1.8 ± 0.8^#^	11.9 ± 4.0^#^	10.0 ± 1.6^#^	1.2 ± 0.4^#^	10.6 ± 2.9^#^
NWSSJD+CPA group	17.4 ± 4.6^#^	4.6 ± 1.5^#^	20.7 ± 1.6^#^	12.6 ± 2.4^#^	2.4 ± 0.5^#^	16.2 ± 4.0^#^	11.4 ± 1.1^#^	2.0 ± 1.2^#^	14.6 ± 8.8^#^	8.8 ± 1.3^#^	0.8 ± 0.8^#^	7.2 ± 7.2^#^
CPA group	10.0 ± 1.6^∗^	11.2 ± 1.9^∗^	52.7 ± 7.5^∗^	8.0 ± 1.6^∗^	7.0 ± 0.7^∗^	47.0 ± 5.3^∗^	5.8 ± 0.8^∗^	7.2 ± 1.5^∗^	55.0 ± 7.8^∗^	4.8 ± 0.8^∗^	4.2 ± 0.8^∗^	46.6 ± 6.2^∗^

Note: compared with control, ^∗^*P* < 0.05; compared with CPA, ^#^*P* < 0.05.

## Data Availability

The data used to support the findings of this study are available from the corresponding author upon request.
